# Prediction of high airway pressure using a non-linear autoregressive model of pulmonary mechanics

**DOI:** 10.1186/s12938-017-0415-y

**Published:** 2017-11-02

**Authors:** Ruby Langdon, Paul D. Docherty, Christoph Schranz, J. Geoffrey Chase

**Affiliations:** 1 Department of Mechanical Engineering, University of Canterbury, Private bag 4800, Christchurch, 8140 New Zealand; 2 Hamilton Medical, Via Crusch 8, 7402 Bonaduz, Switzerland

**Keywords:** Pulmonary modelling, Autoregressive models, Biomedical systems

## Abstract

**Background:**

For mechanically ventilated patients with acute respiratory distress syndrome (ARDS), suboptimal PEEP levels can cause ventilator induced lung injury (VILI). In particular, high PEEP and high peak inspiratory pressures (PIP) can cause over distension of alveoli that is associated with VILI. However, PEEP must also be sufficient to maintain recruitment in ARDS lungs. A lung model that accurately and precisely predicts the outcome of an increase in PEEP may allow dangerous high PIP to be avoided, and reduce the incidence of VILI.

**Methods and results:**

Sixteen pressure-flow data sets were collected from nine mechanically ventilated ARDs patients that underwent one or more recruitment manoeuvres. A nonlinear autoregressive (NARX) model was identified on one or more adjacent PEEP steps, and extrapolated to predict PIP at 2, 4, and 6 cmH_2_O PEEP horizons. The analysis considered whether the predicted and measured PIP exceeded a threshold of 40 cmH_2_O. A direct comparison of the method was made using the first order model of pulmonary mechanics (FOM(I)). Additionally, a further, more clinically appropriate method for the FOM was tested, in which the FOM was trained on a single PEEP prior to prediction (FOM(II)). The NARX model exhibited very high sensitivity (> 0.96) in all cases, and a high specificity (> 0.88). While both FOM methods had a high specificity (> 0.96), the sensitivity was much lower, with a mean of 0.68 for FOM(I), and 0.82 for FOM(II).

**Conclusions:**

Clinically, false negatives are more harmful than false positives, as a high PIP may result in distension and VILI. Thus, the NARX model may be more effective than the FOM in allowing clinicians to reduce the risk of applying a PEEP that results in dangerously high airway pressures.

## Background

Acute respiratory distress syndrome (ARDS) requires mechanical ventilation (MV) in the intensive care unit (ICU). ARDS can involve elements of inflammation in the lungs and fluid accumulation in airways, and has a mortality rate of 46% [1]. Positive end-expiratory pressure (PEEP) is an important ventilator setting used to prevent de-recruitment of lung units at the end of expiration [2, 3]. However, when PEEP levels are too high, over distension of alveoli may cause ventilator induced lung injury (VILI) [4]. Whenever damage is caused to the lung tissue, there is a release of biological mediators, which can lead to organ failure and increased mortality [5]. Hence, setting PEEP levels to maintain recruited alveoli, while avoiding distension, is necessary to minimise VILI and improve outcomes [6–9].

Physiological modelling can capture patient-specific pulmonary mechanics and aid determination of optimal ventilator settings for each patient [10–14]. We have previously proposed a nonlinear autoregressive (NARX) model of respiratory mechanics [15, 16]. The model uses basis functions to capture pulmonary elastance as a function of airway pressure, and multiple time-dependent resistance coefficients to capture impedance and viscoelastic effects. The NARX model has successfully captured airway pressure waveforms in ARDS patients during recruitment manoeuvres (RM) [15], and successfully predicted airway pressure at higher PEEP [16].

High airway pressure (> 40 cmH_2_O) due to excessive PEEP can contribute to over-distension and VILI [17–20]. Thus, limited pressure, as well as low tidal volume and other parameters, is considered lung protective [21]. Using prediction to avoid PEEP levels that induce high PIP may reduce the incidence of VILI, and the associated mortality and morbidity. Such prediction would provide clinicians with an indication of the risk associated with an increase in PEEP and a further metric to aid decision support. The aim of this work is to assess the ability of the NARX model to predict the high pressure that may cause pulmonary distension in ARDS patients, where the prediction horizon is a PEEP step increase of 2, 4, or 6 cmH_2_O. A comparison is made with the well-known and frequently used first order model (FOM) [6], which, in contrast with the NARX model, uses a single elastance term.

## Methods

### Data

This analysis uses data from a pilot Clinical Utilisation of Respiratory Elastance (CURE) software trial [22]. Airway pressure and flow data were collected from 10 fully sedated ARDS patients, of which seven were ventilated in pressure controlled mode, and three in volume controlled mode. Ethics approval for this study and subsequent use of collected data was granted by the New Zealand South Regional Ethics Committee. Informed consent was obtained from the patients. Patient age ranged from 18 to 88, with a mean of 55 years. The mean breathing rate was approximately 18 breaths/min, with no end-inspiratory pause. Pressure and flow were recorded from a Puritan Bennett 840 ventilator at a sampling rate of 50 Hz. Volume was calculated from continuous integration of the flow, with compensation for volume drift to maintain a volume of 0 mL at PEEP.

Patients underwent one or more RMs with initial PEEP = 8–16 cmH_2_O. PEEP was then increased in steps of 2 cmH_2_O after approximately 10 breaths at each PEEP level. The RMs used differing numbers of PEEP steps, and reached different PIP levels. The data sets contained a range of 5–9 PEEP steps. It is also important to note these patients were in the middle of care and PEEP = 16 cmH2O is not unreasonable for some patients. If the patient is not responding at this level a RM is an acceptable course of action, leading to the wider range of PEEP at RM commencement in this data. One of the ten patients was excluded as they were not ventilated to a PIP of 40 cmH_2_O. Of the nine remaining patients, three underwent multiple RMs during the trial. In total, there were 16 data sets available for analysis.

### Respiratory models

The first order model (FOM) of pulmonary mechanics forms the basis of the NARX model. The FOM contains single resistive and elastic components:

1 $$P\left( t \right) = R\dot{V}\left( t \right) + EV\left( t \right) + P_{0} \left( t \right)$$

where *P* is the measured airway pressure (cmH_2_O), *t* is time (s), *R* is the airway resistance (cmH_2_O s/L), $\dot{V}$ is the airway flow rate (L/s), *E* is the elastance of the lung tissue and chest wall (cmH_2_O/L), *V* is the air volume in the lung above volume at PEEP (L), and *P*_0_ is the offset pressure (cmH_2_O).

The NARX model builds upon the FOM by incorporating pressure dependent basis functions to describe elastance, and multiple time dependent terms that represent the effect of airway resistance to flow and changes in flow. The NARX model is defined [15]:

2 $$P\left( t \right) = \mathop \sum \limits_{i = 1}^{M = 4} E_{i} \phi_{i} \left( {P\left( t \right)} \right)V\left( t \right) + \mathop \sum \limits_{j = 0}^{L = 170} R_{j} \dot{V}\left( {t_{ - j} } \right) + P_{0} \left( t \right)$$

where *ϕ*_*i*_(*P*(*t*)) is the particular basis function value for a given pressure measurement (dimensionless); *E*_*i*_ is the elastance coefficient for a given basis function (cmH_2_O/L), *M* is the number of basis functions used, *R*_*j*_ is a series of terms that capture resistance and inertance of the flow (cmH_2_O s/L); *L* is the number of resistive terms. The subscript − *j* in the second term refers to the previous time samples. Thus, each *P*(*t*) is calculated from information from the previous 170 data points. The sum of the basis functions multiplied by their *E*_*i*_ coefficients represent elastance as a function of pressure. The FOM can be replicated with *M* = *L* = 1, and zero order basis functions.

**Fig. 1:**
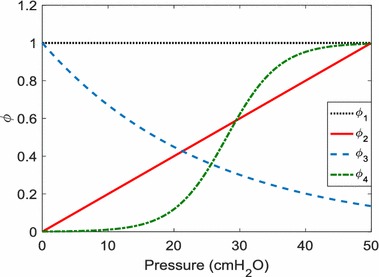
Constant, exponential, linear, and sigmoidal basis functions

Figure 1 shows the four basis functions used to describe elastance. These functions were selected previously [16] as a linear combination of these shapes were able to capture the range of elastance profiles observed in a larger patient cohort:

3a $$\phi_{1} = 1$$

3b $$\phi_{2} = \frac{P}{50}$$

3c $$\phi_{3} = e^{ - 0.04P}$$

3d $$\phi_{4} = \frac{1}{{1 + e^{{ - 0.25\left( {P - 28} \right)}} }}$$

To identify patient-specific models, the non-linear elastance terms are decomposed into linear terms using Eqs. 3a, 3b, 3c, 3d and a linear system is evaluated across certain subsets of the full data range (*t*_*0*_ − *t*_*N,*_ where *N* < *N*_*D*_ and *N*_*D*_ is the length of the full data set). This subset of data is the ‘training set’.

4 $${\mathbf{Ax}} = {\mathbf{b}}$$

where:

4a $${\mathbf{A}} = \left[ {\begin{array}{*{20}c} {\phi_{1} \left( {P\left( {t_{0} } \right)} \right)V\left( {t_{0} } \right)} & \cdots & {\phi_{M} \left( {P\left( {t_{0} } \right)} \right)V\left( {t_{0} } \right)} & {\dot{V}\left( {t_{0} } \right)} & \cdots & {\dot{V}\left( {t_{ - L} } \right)} \\ {\phi_{1} \left( {P\left( {t_{1} } \right)} \right)V\left( {t_{1} } \right)} & \cdots & {\phi_{M} \left( {P\left( {t_{1} } \right)} \right)V\left( {t_{1} } \right)} & {\dot{V}\left( {t_{1} } \right)} & \cdots & {\dot{V}\left( {t_{1 - L} } \right)} \\ \vdots & \vdots & \vdots & \vdots & \vdots & \vdots \\ {\phi_{1} \left( {P\left( {t_{N} } \right)} \right)V\left( {t_{N} } \right)} & \cdots & {\phi_{M} \left( {P\left( {t_{n} } \right)} \right)V\left( {t_{N} } \right)} & {\dot{V}\left( {t_{N} } \right)} & \cdots & {\dot{V}\left( {t_{N - L} } \right)} \\ \end{array} } \right]$$

4b $${\mathbf{b}} = \left[ {\begin{array}{*{20}c} {P\left( {t_{0} } \right) - P_{0} \left( {t_{0} } \right)} \\ {P\left( {t_{1} } \right) - P_{0} \left( {t_{1} } \right)} \\ \vdots \\ {P\left( {t_{N} } \right) - P_{0} \left( {t_{N} } \right)} \\ \end{array} } \right];\;\;{\text{and}}\;\;{\mathbf{x = }}\left[ {\begin{array}{*{20}c} {E_{1} } \\ \vdots \\ {E_{M} } \\ {R_{1} } \\ \vdots \\ {R_{L} } \\ \end{array} } \right]$$

The linear system can be evaluated via matrix psuedo inverse to provide **x**.

### Analysis

This analysis compares NARX and FOM predictions for the occurrence of PIP > 40 cmH_2_O at PEEP levels higher than the training set. This pressure threshold was arbitrarily chosen, but is generally considered an upper bound to acceptable ventilation pressures for critically ill ARDS patients. True positive, true negative, false positive, and false negative results were recorded for both models. The sensitivity and specificity were calculated. Receiver operating characteristic (ROC) curves were generated by varying the discrimination threshold.

For each RM, the NARX model was first trained on the lowest PEEP level (PEEP_min_) in the data. The basis functions that formed the patient model were extrapolated to predict the PIP at PEEP_min_ + {2, 4, 6} cmH_2_O, respectively. The predicted PIP was compared with the measured PIP at these higher PEEP levels. Then the PEEP_min_ and PEEP_min_ + 2 cmH_2_O levels were used as the training set, and PIP was predicted at PEEPmin + {4, 6, 8} cmH_2_O. The training set was enlarged to incorporate more PEEP levels until there was only a single PEEP step left for prediction.

**Fig. 2:**
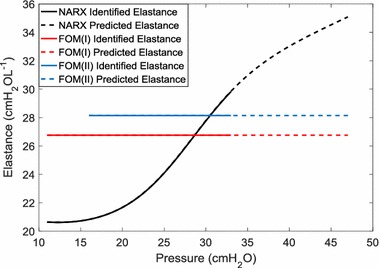
Identified elastance and predicted elastance for a particular ARDS patient

The predictive ability of the FOM was used as a comparator for the NARX. The FOM is generally considered to be most relevant over small pressure ranges. FOM(I), uses the same training set as the NARX model, FOM(II) uses the elastance determined from a single PEEP level to predict PIP at one to three PEEP steps above that level. FOM(I) allows a direct comparison with the NARX model method, and FOM(II) allows the FOM to operate in a scenario more suited to its strengths. Figure 2 shows one patient’s identified and extrapolated elastance for the NARX and FOM methods.

## Results

**Fig. 3:**
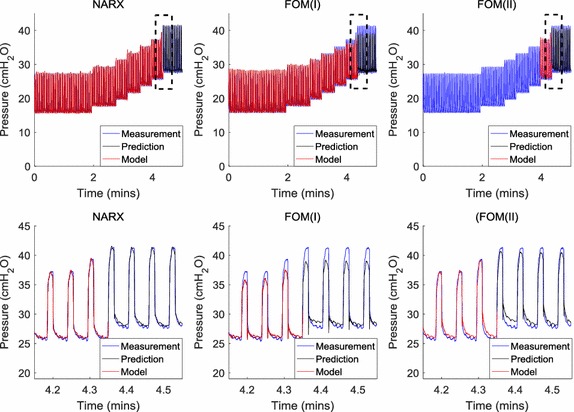
Prediction and training data. Bottom figures are zoomed into the corresponding box shown in the top figures

Figure 3 shows prediction results for one RM. In this particular case, the NARX and FOM(I) models were identified on six PEEP steps and extrapolated to the seventh PEEP step. The NARX model provided an accurate prediction of the pressure waveform and correctly determined that peak pressure at the seventh PEEP step would be greater than 40 cmH_2_O. The FOM(I) prediction was substantially lower than the true peak pressure at the seventh PEEP step and gave a false negative result. FOM(II) was able to correctly predict that peak pressure would exceed 40 cmH_2_O at the seventh PEEP step. However, the predicted waveform had larger residuals compared to the NARX model.

Table 1 shows the confusion matrices for the NARX model, FOM(I), and FOM(II) at each prediction horizon. The NARX model sensitivity was substantially better than the FOM(I) or FOM(II) in all cases. As expected, FOM(II) was more sensitive than FOM(I). The specificity of the NARX model was lower than FOM(I) and FOM(II). Note that there is a general, but imperfect, trend for the prediction metric scores to be poorer as the prediction PEEP moves further from the training PEEP.

**Table 1:** Prediction results

		Predicted *P* > 40 cmH_2_O	Predicted *P* < 40 cmH_2_O	
NARX	Measured *P* > 40 cmH_2_O	TP [45, 43, 42]	FN [0, 2, 1]	Sensitivity [1.00, 0.96, 0.98]
	Measured *P* < 40 cmH_2_O	FP [3, 3, 5]	TN [54, 36, 24]	Specificity [0.95, 0.88, 0.89]
FOM(I)	Measured *P* > 40 cmH_2_O	TP [31, 30, 29]	FN [14, 14, 15]	Sensitivity [0.69, 0.67, 0.67]
	Measured *P* < 40 cmH_2_O	FP [0, 0, 1]	TN [57, 41, 26]	Specificity [1.00, 1.00, 0.96]
FOM(II)	Measured *P* > 40 cmH_2_O	TP [39, 38, 32]	FN [6, 7, 11]	Sensitivity [0.87, 0.84, 0.74]
	Measured *P* < 40 cmH_2_O	FP [0, 0, 1]	TN [57, 41, 26]	Specificity [1.00, 1.00, 0.96]

Brackets denote the classifications for the 2, 4, and 6 cmH_2_O prediction horizons

**Fig. 4:**
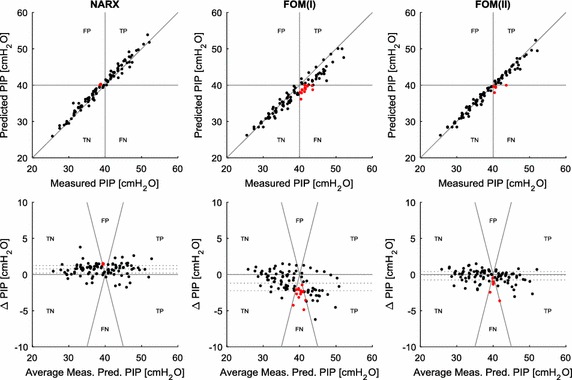
Relationship between measured and predicted peak pressures for the models analysed. Top—correlations; bottom—Bland–Altman

The relationship between measured and predicted peak pressures across the cohort is given in Fig. 4, for the prediction a single PEEP step up from the training data. The three cases of false positive detection by the NARX model occurred when the measured PIP was very close to 40 cmH_2_O. In all three of these cases, measured PIP was ≥ 38.6 cmH_2_O, indicating the NARX model prediction was very close to the clinical outcome. In the Bland–Altman plots, dotted lines give the 25th, 50th, and 75th percentiles of the difference in measured and predicted PIP. There is a slight bias in the NARX model for predictions to be higher than measured. FOM(I) has a relatively large bias towards low predictions, and FOM(II) is also slightly biased low.

**Fig. 5:**
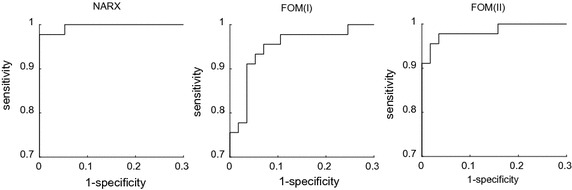
ROC curves for each model

The ROC curves in Fig. 5, for a single PEEP step prediction, plot the true positive rate against the false positive rate as the discrimination threshold was varied (Fig. 5). While all models performed significantly better than a random classifier, the area under the curve was clearly greatest for the NARX model, indicating the best overall performance. The optimal threshold was 40.3 cmH_2_O for the NARX model, 37.8 cmH_2_O for FOM(I), and 39.3 cmH_2_O for FOM(II). The optimal threshold was calculated as the point on the ROC curve closest to (0, 1). Calculating the Youden index yielded the same results.

## Discussion

Clinically, false negatives are much more harmful to patients than false positives. In the context of pulmonary distension, a false negative may result in higher ventilation pressures that lead to distension and VILI. In contrast, a false positive result may mean clinicians do not increase PEEP, and miss out on the potential for improved recruitment at the higher PEEP level. Hence, the comparative impact of a false negative is stronger than the impact of a false positive, and thus, a high sensitivity should be favoured when predicting the outcomes of potential treatment methods.

Elastance captures the static tidal pressure required for a given volume of inspired air. While PEEP is the model input that allows the model to fit to the pressure troughs in each breath, an accurate elastance allows an accurate model fit to PIP. If predicted elastance is too high, a predicted breath will have a PIP that is higher than the true PIP. Similarly, if predicted elastance is too low, the predicted PIP will be lower than the true PIP.

VILI often occurs at the alveoli, due to mechanical strain caused by high alveolar pressure [23]. Due to low flowrates in the lower bronchiole close to the alveoli and the compliance of the bronchial path, the PIP measured at the airway is generally higher than the pressure experienced at the alveoli. Since airway resistance reduces at high pressures, higher than expected PIP levels are more likely due to increases in elastance at high pressure. Such an outcome is indicative of alveolar distension. Although this clinical protocol did not utilise the end-inspiratory pause needed to approximate alveolar pressure, it utilised a proxy metric (PIP) that is easily available in typical clinical practice.

In this analysis, there were no false negative and only three false positive predictions from the NARX model over a prediction horizon of one PEEP step. In the three false positive cases, the mean difference between measured and predicted peak pressures was only 1.5 cmH_2_O. For the NARX false positives that occurred at prediction horizons of two and three PEEP steps, the mean difference between measured and predicted PIP was 2.4 cmH_2_O in both cases. Thus, the false positives did not actually represent poor prediction. However, the incidence of these false positives may imply that the basis function shapes slightly overestimate the onset of distension.

FOM(I) and FOM(II) were unable to always predict when measured PIP was likely to be greater than 40 cmH_2_O (Table 1). The FOM assumes a constant elastance (Eq. 1). In reality, elastance changes during inflation according to recruitment and distension effects [24, 25]. In general, distension effects mean that elastance is likely to be higher at pressure close to 40 cmH_2_O than at lower pressures, as most of the possible recruitment has already been achieved. Thus, in a situation with increasing elastance with PEEP, the single elastance term identified in the FOM will lead to underestimated PIP. This led to the high rate of false negatives given by FOM(I) and FOM(II). In contrast, the extrapolation of the NARX elastance shape to higher pressures enables the prediction of a higher elastance at higher PEEP, and thus very few false negatives occurred.

FOM(II) uses identification data from only one PEEP step previous to the prediction PEEP. This method better represented the clinical use of the FOM. While less data was used for parameter identification in comparison to FOM(I), FOM(II) was more successful at predicting PIP. Compared to FOM(I) the sensitivity of FOM(II) increased by an average of 0.14 percentage points for the three prediction horizons. This occurred because the single elastance of FOM(II) did not need to partially represent the lower elastance of the lower pressure ranges (Fig. 2). However, the predicted peak pressures of FOM(II) tended to be lower than the NARX prediction and had higher residuals (Fig. 3).

The FOM(I) and FOM(II) specificities were higher than the NARX model specificities for each prediction horizon. This was an expected result, as a FOM false positive would only occur if elastance was decreasing when PIP was near 40 cmH_2_O. When peak pressure is close to 40 cmH_2_O, elastance is very unlikely to decrease as all recruitable lung volumes are likely to be recruited at lower pressures. Alternatively, FOM false positives would be likely to occur in a decreasing PEEP scenario. A single elastance identified from high pressure data is likely to be too high for an accurate prediction when PEEP is decreased. In this scenario, the NARX would be expected to perform better than the FOM, due to the ability to easily extrapolate a continuous elastance to lower pressures.

The ROC curve analysis (Fig. 5) shows the possible diagnostic equivalence of the models as their diagnostic thresholds are varied. The area under the ROC curve for the NARX model was the largest and yielded a peak sensitivity and specificity of 0.98 and 1.00 when a threshold of 40.3 cmH_2_O is used. Since the optimum threshold of the NARX is very close to 40 cmH_2_O, the NARX predicted pressures were very precise in the region of clinical interest. The FOM(I) yielded a maximum diagnostic equivalence at 37.8 cmH_2_O and had a sensitivity of 0.96 and specificity of 0.93 at this point. The FOM(II) optima occurred at 39.3 cmH_2_O and had a sensitivity of 0.98 and a specificity of 0.96 at this point. While the NARX precision and accuracy exceeded the performance of the FOM, it should be noted that the performance of all models would be acceptable within the variance expected in clinical practice.

Clinically, once a dangerous PIP has been recorded, PEEP would not be increased further as the risk of distension and VILI would be high. Thus, outside of an initial recruitment manoeuvre, PEEP might not be increased to allow peak pressures beyond the clinician’s chosen threshold. In this analysis, we included all available data from the recruitment manoeuvres and thus contradicted the clinical process. However, this was necessary to establish that the models did not erroneously predict a low PIP at high PEEP. Furthermore, ceasing the evaluation at the first instance of PIP > 40 cmH_2_O would generate an incorrect low true positive rate, and thus the sensitivity calculation would be negatively affected.

In this analysis we used data from seven patients on pressure controlled ventilation and three patients on volume controlled ventilation. In pressure controlled data, the PIP is a setting defined by the clinician, and thus it may seem strange to analyse the ability of the model to predict PIP in pressure controlled mode. However, none of the modelling approaches used in this analysis incorporated a priori information on the applied ventilator settings. In contrast, the modelling approaches provide a transfer function between pressure and flow. Thus the ability to predict pressure from flow data remains scientifically valid, even in pressure controlled mode, when the model does not use the ventilator settings as an input. Furthermore, parameterisation must be considered when model residuals are used to assess model performance [26]. In particular, in such cases extraneous parameters could enable improved fitting, but they could also confound the precise identification of physiologically meaningful parameters and thus limit the applicability of the model for prediction or extrapolation of behaviour. However, the level of model parameterisation is insignificant when the ability to precisely extrapolate beyond the identification domain is used to assess model suitability.

This model was designed specifically to capture non-linear elastance effects and uses pressure dependent elastance terms. However, we note that this formulation, like all models has limitations in certain behaviours. In particular, the model may well not perform well in some clinical cases, such as atelectasis or high auto-PEEP. However, the primary goal of this research was determine the model’s ability to predict the likelihood of exceeding a particular PIP threshold. Hence, it was critical to be able to extrapolate elastance beyond the pressure in the identification sets. Pressure and volume dependent elastance formulation are mathematically equivalent due to the generally monotonic behaviour of pressure and volume. However, integration of flow signals yields drift in volume values that can confound PIP prediction. Pressure signals can be more effectively calibrated across PEEP steps and are thus a much more stable parameter to extrapolate elastance.

While the cohort is representative of modern ICU patients, the sample size of nine patients and 16 RMs is relatively small. The method should be tested on a larger cohort under varying disease states and ventilation modes to confirm the results. Additionally, the threshold pressure of 40 cmH_2_O was somewhat arbitrarily chosen. While this pressure would normally be considered high, it may not necessarily cause over-distension in all patients. However, the findings of this analysis imply that the NARX model’s variable elastance can enable accurate and precise PIP predictions at any pressures thresholds that are chosen with the intention to limit distension.

The NARX model predicted high peak pressures more accurately than the FOM, and importantly, had very low instances of false negatives. Zero false negatives occurred at the prediction horizon of one PEEP step. The NARX model may aid clinicians in deciding whether to raise the PEEP setting for individual patients, and avoid dangerous PEEP levels that may cause distension and VILI.

## Conclusions

The NARX model was more successful than the FOM at predicting high airway pressure (Fig. 4). The NARX model had very few instances of false negative results, while the FOM frequently failed to predict when PIP would exceed 40 cmH_2_O (Table 1). While FOM(II) improved the FOM sensitivity over FOM(I), it was still less successful than the NARX model. In contrast, the NARX model yielded a limited number of false positive predictions, though the specificity of the NARX model remained relatively high at 0.95, 0.88, and 0.89 for the 2, 4, and 6 cmH_2_O prediction horizons, respectively. The error in the NARX false positive predictions was small, and the consequences of false positives are far less severe than the potential VILI that may result from false negative predictions. Thus, the NARX model predictive capability has the potential to reduce the risk of patients being ventilated at dangerously high PEEP levels.

## Abbreviations

ARDS: acute respiratory distress syndrome
FOM: first order model
ICU: intensive care unit
MV: mechanical ventilation
NARX: nonlinear autoregressive
PEEP: positive end expiratory pressure
PIP: peak inspiratory pressure
RM: recruitment manoeuvre
VILI: ventilator induced lung injury
